# Author Correction: Evolution and expression patterns of the neo-sex chromosomes of the crested ibis

**DOI:** 10.1038/s41467-024-53229-x

**Published:** 2024-10-28

**Authors:** Lulu Xu, Yandong Ren, Jiahong Wu, Tingting Cui, Rong Dong, Chen Huang, Zhe Feng, Tianmin Zhang, Peng Yang, Jiaqing Yuan, Xiao Xu, Jiao Liu, Jinhong Wang, Wu Chen, Da Mi, David M. Irwin, Yaping Yan, Luohao Xu, Xiaoping Yu, Gang Li

**Affiliations:** 1 College of Life Sciences, Shaanxi Normal University, Xi’an, China; 2 MOE Key Laboratory of Freshwater Fish Reproduction and Development, School of Life Sciences, Southwest University, Chongqing, China; 3 Research Center for Qinling Giant Panda, Shaanxi Academy of Forestry, Xi’an, China; 4 Guangzhou Wildlife Research Center, Guangzhou Zoo, Guangzhou, China; 5 Xi’an Haorui Genomics Technology Co., LTD, Xi’an, China; 6 Department of Laboratory Medicine and Pathobiology, University of Toronto, Toronto, ON Canada

**Keywords:** Sexual selection, Evolutionary biology, Genome, Evolutionary genetics

Correction to: *Nature Communications* 10.1038/s41467-024-46052-x, published online 23 February 2024

The original version of this Article contained an error in the third sentence of the third paragraph of the Results, which incorrectly read “We annotated 414 protein-coding genes on the sexually differentiated region (SDR) of chromosome W, making it the most gene-rich SDR of bird W chromosomes, suggesting that only 54.6% of the original gene content was lost from the W chromosome, in contrast to the greater that 90% found in most land birds.”. The correct version states “We annotated 414 protein-coding genes on the sexually differentiated region (SDR) of chromosome W, making it the most gene-rich SDR of bird W chromosomes. After removing genes that replicated independently on the W chromosome, 83.4% of the original gene content was lost from the W chromosome, in contrast to the greater than 90% found in most land birds”.

This has been corrected in both the PDF and HTML versions of the Article.

