# Supplementary material for: Evolution and expression patterns of the neo-sex chromosomes of the crested ibis
Source: Nat Commun. 2024 Feb 23;15:1670. doi: 10.1038/s41467-024-46052-x (PMC10891136; doi:10.1038/s41467-024-46052-x)
Supplement: Supplementary file 1 — Supplementary Information [file 41467_2024_46052_MOESM1_ESM.pdf]

Evolution and expression patterns of the neo-sex chromosomes of the crested ibis

Lulu Xu[1], Yandong Ren[1], Jiahong Wu[2], Tingting Cui[1], Rong Dong[3], Chen Huang[1], Zhe Feng[1], Tianmin Zhang[1], Peng Yang[1], Jiaqing Yuan[1], Xiao Xu[1], Jiao Liu[2], Jinhong Wang[1], Wu Chen[4], Da Mi[5], David M. Irwin[6], Yaping Yan[1], Luohao Xu[2]*, Xiaoping Yu[1]*, Gang Li[1,4]*

*Corresponding author. E-mails: gli@snnu.edu.cn; yuxp64@163.com; luohaox@gmail.com

This Supplementary Information includes:

Supplementary Information includes:

Supplementary Figures 1-17

Supplementary Tables 1-7

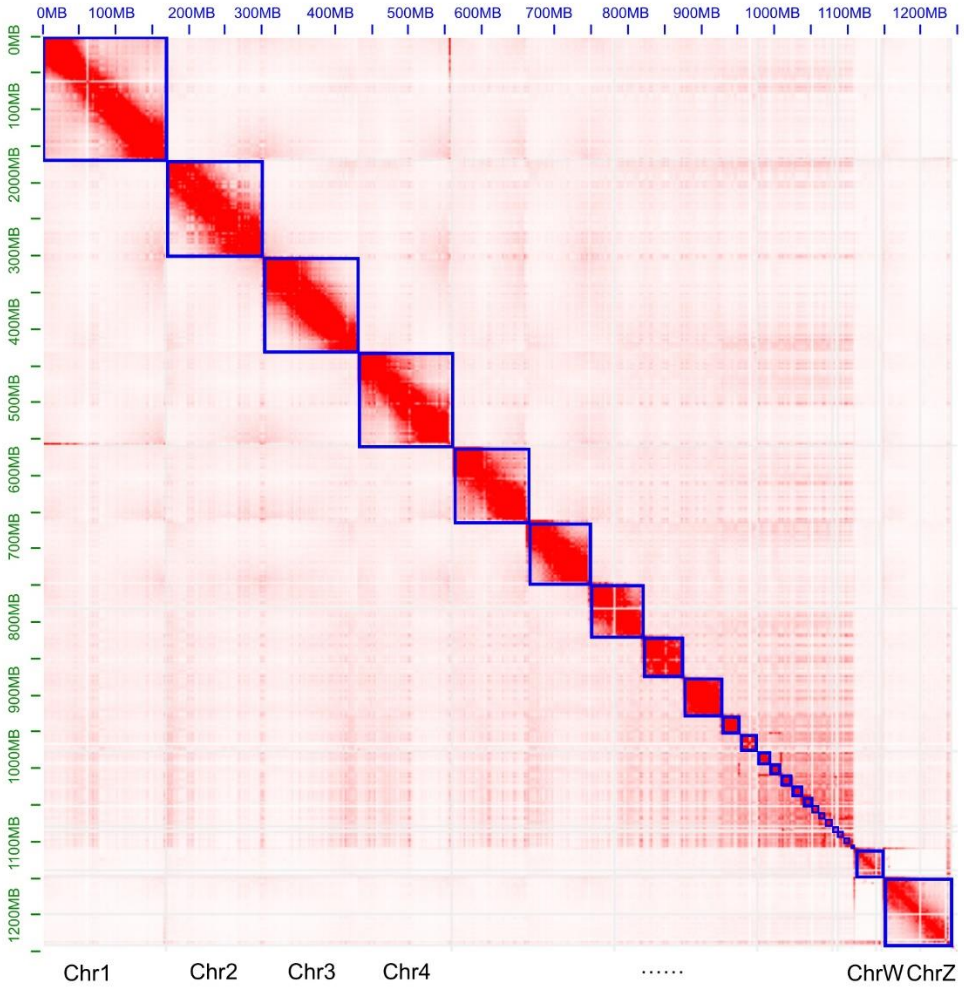

Supplementary Fig. 1 Chromosome-level assembly of crested ibis. The Hi-C map shows genome-wide interactions visualized by Juicer. The smaller autosomes are not labelled due to the space constraints in the figure.

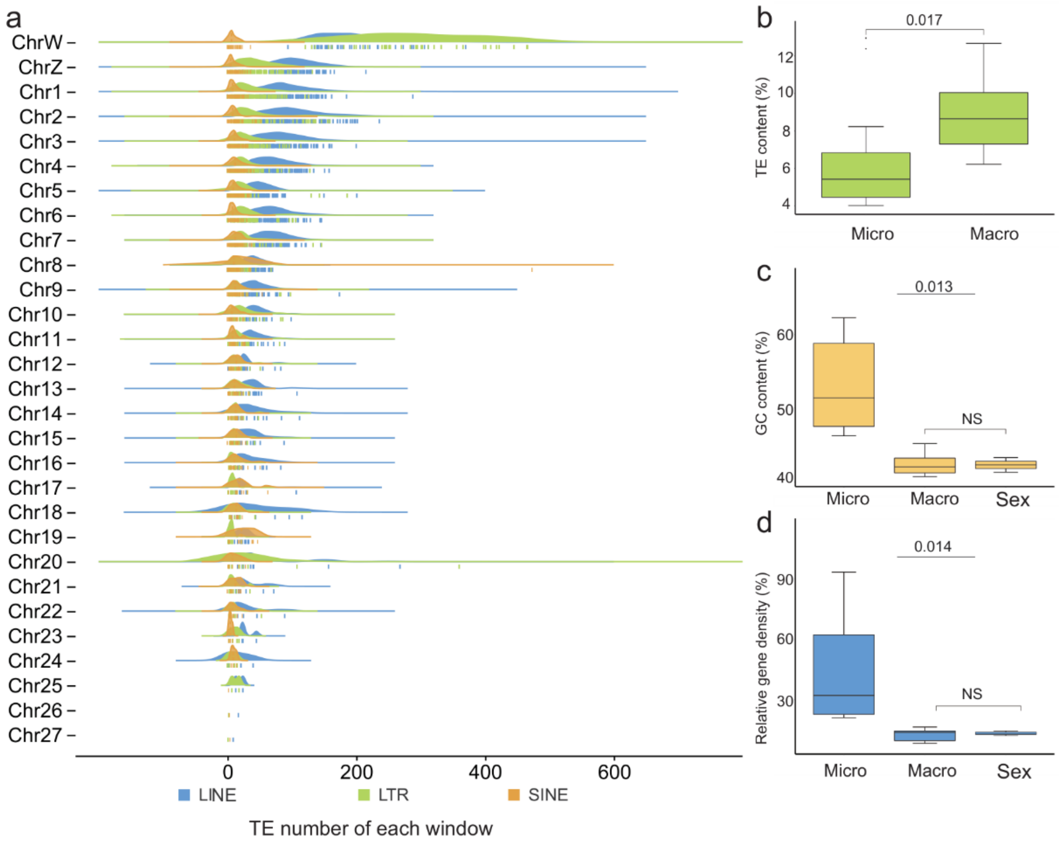

Supplementary Fig. 2 Different landscape of macro- micro- and sex chromosomes. **a** Ridgeline plots display the TEs abundance of each chromosome. X axis is TE number of each window on each chromosome, the window size is 100kb. The height of mountain shows the number of windows with same TE density. **b** Comparison of TE content of micro- and macro- chromosomes. Two-sided t-test. **c** Comparison of GC content of different chromosomes. One-way Welch's ANOVA test was used for three groups of data analysis. **d** Comparison of dene density of different chromosomes. One-way Welch's ANOVA test was used for three groups of data analysis. The number on the horizontal line represents p value. NS represents no significant difference. Source data are provided as a Source Data file.

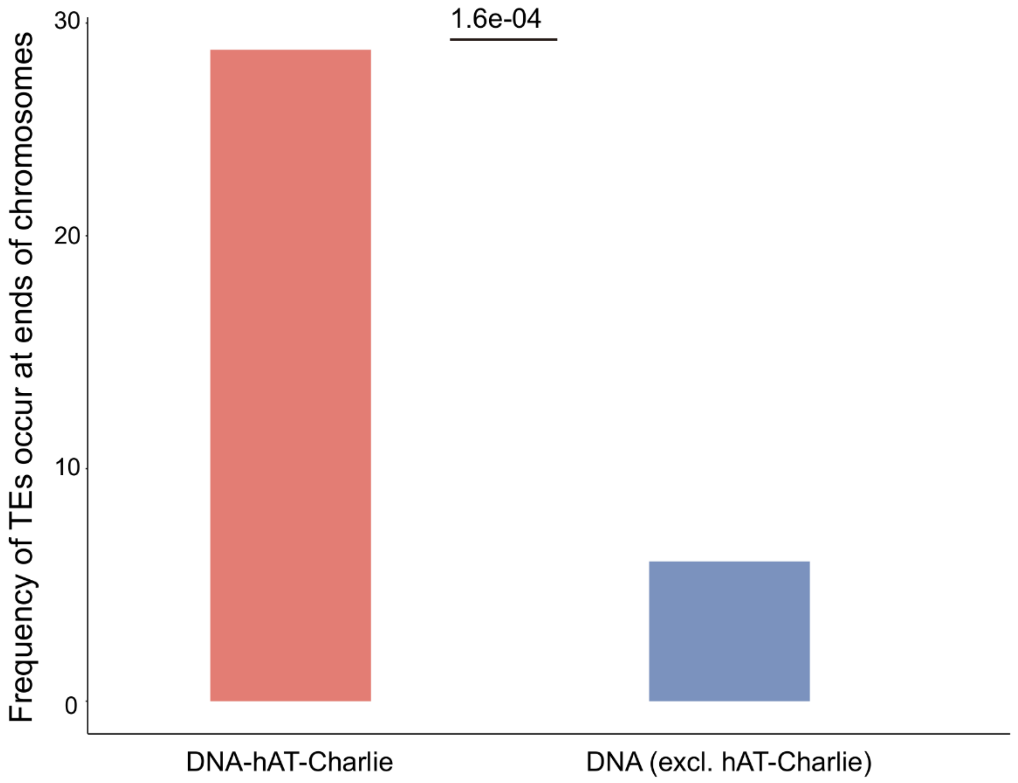

Supplementary Fig. 3 Comparison of frequency of TEs occur at ends of chromosomes. DNA-hAT-Charlie are significantly more occurred at terminuses (refer to 50 kb regions at the ends of chromosomes) of chromosomes than other DNAs of TE. Chi-Squared Test was used for data analysis. The number on the horizontal line represents p value. Source data are provided as a Source Data file.

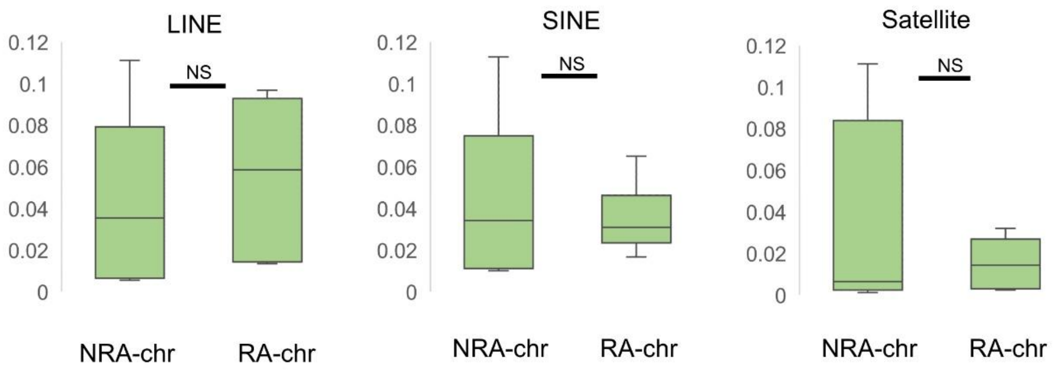

Supplementary Fig. 4 Comparison of ratio of TEs in non- rearranged chromosomes (NRA-chr) and rearranged chromosomes (RA-chr). n =6 and 7 of NRA-chr and RA-chr, respectively. Two-sided T-test was used for data analysis. NS: Not significant. Source data are provided as a Source Data file.

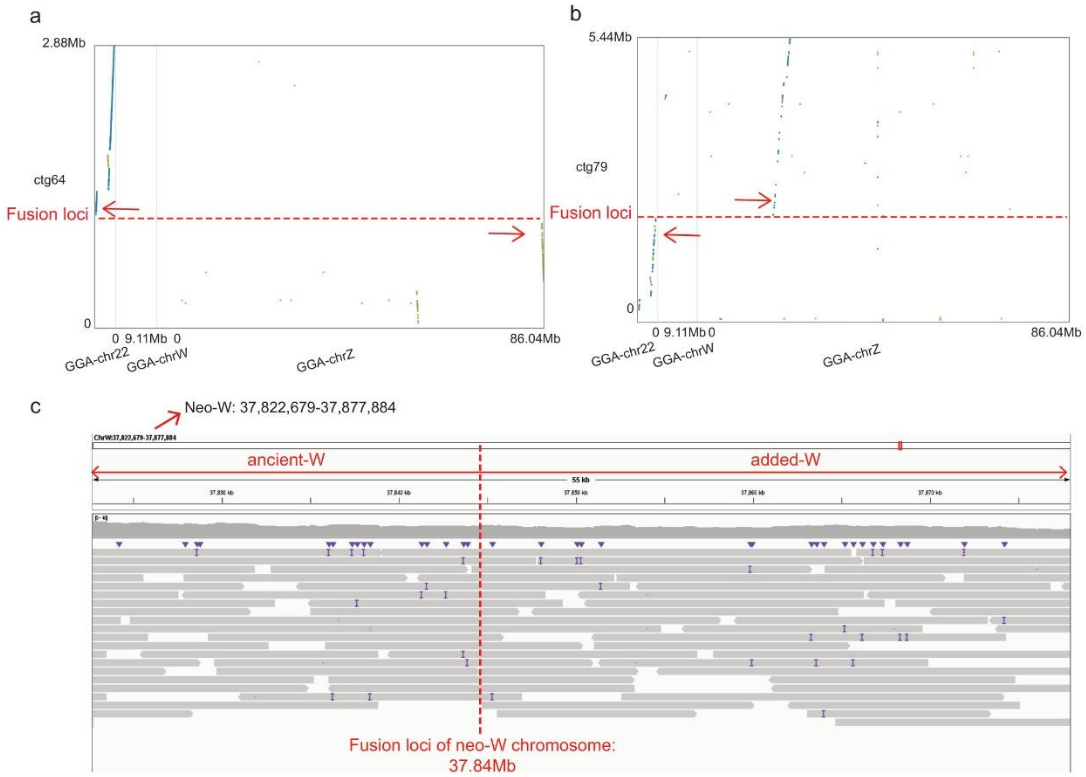

Supplementary Fig. 5 Evidence of neo-sex chromosome formation by a fusion event. a, b the two screenshots show the colinear relationship between two contigs: ctg64 (anchored on neo-Z) and ctg79 (anchored on neo-W). **c** IGV screenshot of the results of raw reads mapping to neo-W. The dashed line indicates the fusion site of neo-W, which is run crossed by consecutive reads.

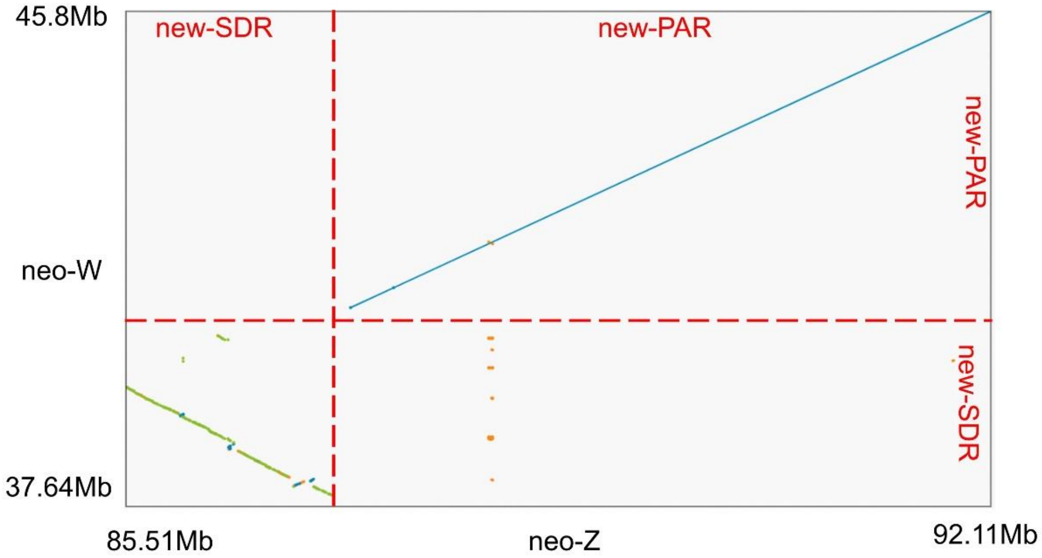

Supplementary Fig. 6 Synteny dot plot of neo-Z and neo-W. The green dots show the inversion which located at the New-SDR. The junction of red dashed lines links the New-SDR and New-PAR.

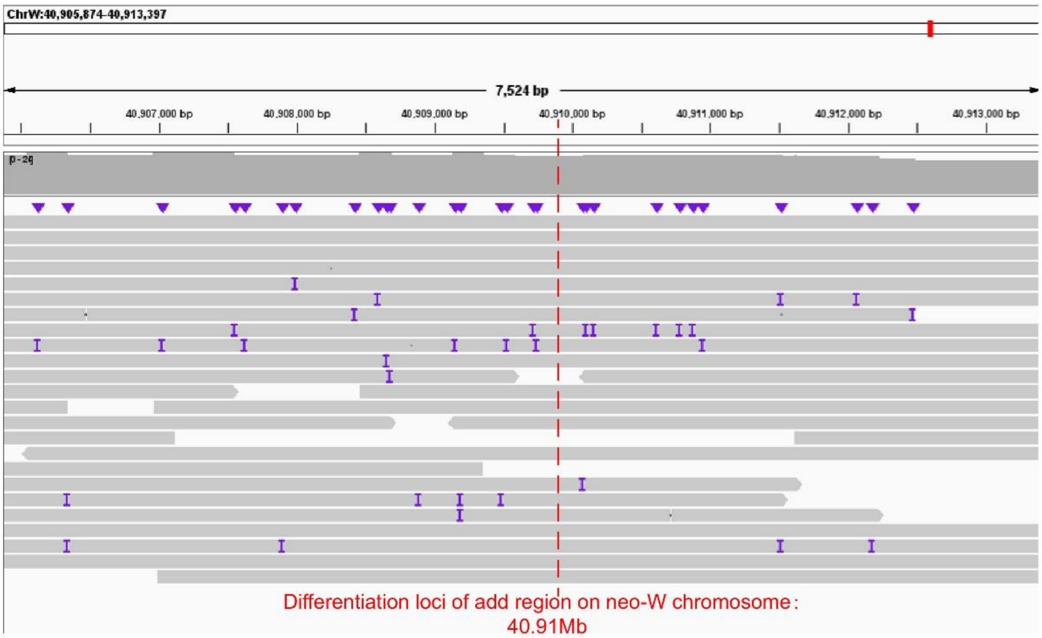

Supplementary Fig. 7 IGV screenshot of the results of raw reads mapping to neo-W. The dashed line indicates the differentiation site of neo-W, which is run crossed by consecutive reads.

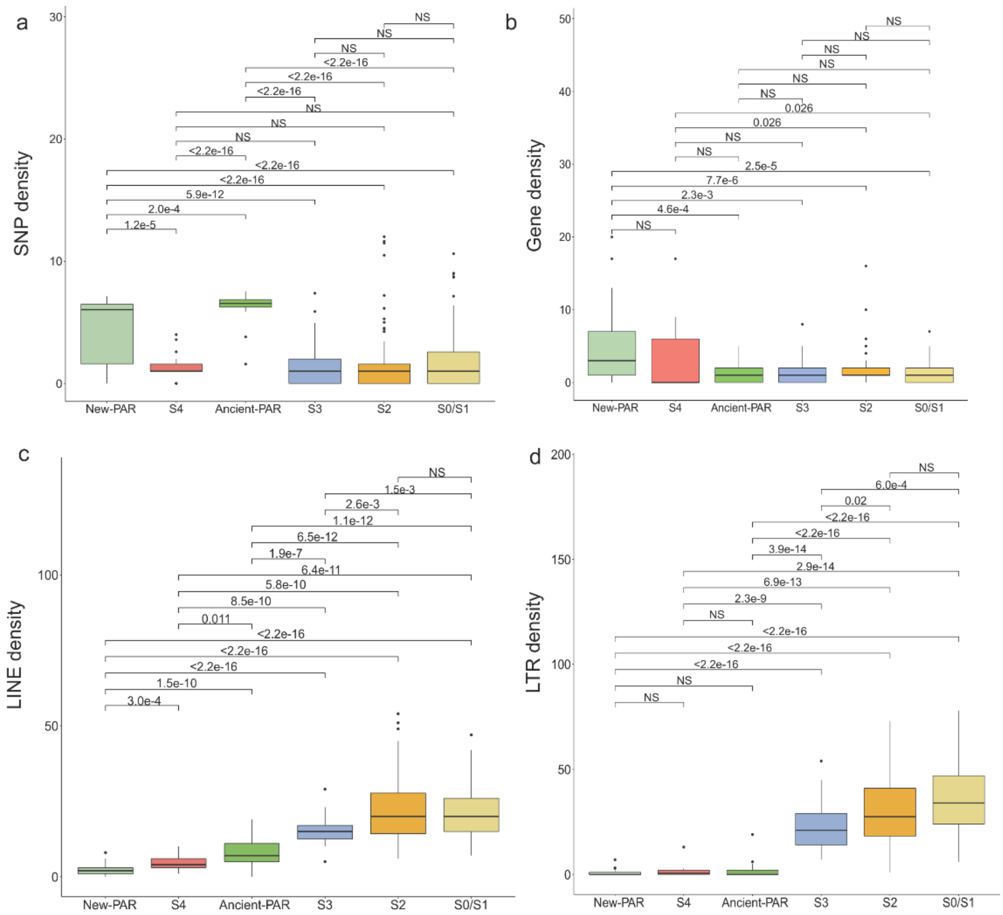

Supplementary Fig. 8 Comparisons of genome characters of each region on neo-W chromosome. a SNP density, **b** gene density, **c** LINE density, **d** LTR density. Two-sided T-test. NS: Not significant. Number on the horizontal line represents p values. Different color represents different region on neo-W. Source data are provided as a Source Data file.

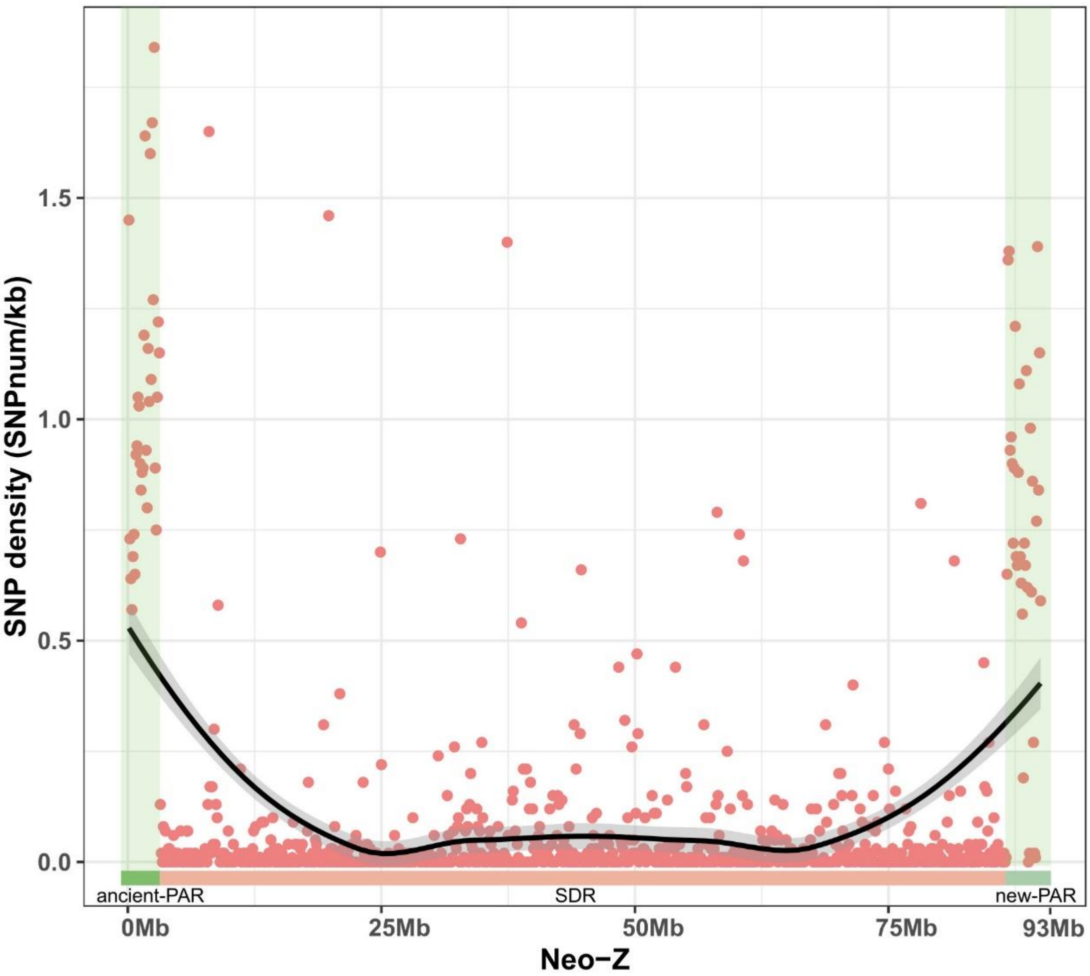

Supplementary Fig. 9 SNP density distribution of neo-Z chromosome. Each dot represents the SNP density of a 10kb-size window. Two PARs located at the tips of Neo-Z chromosome show a higher SNP density than SDR. Source data are provided as a Source Data file.

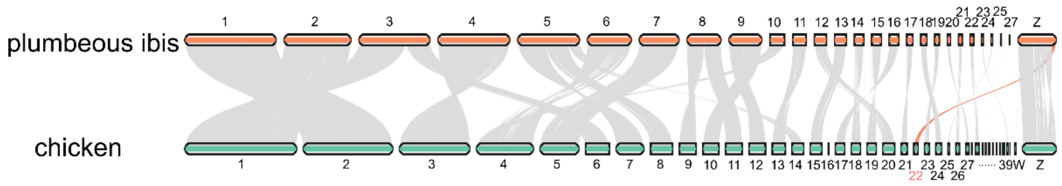

Supplementary Fig. 10 Synteny analysis between chicken and plumbeous ibis. The plumbeous ibis Z chromosome fused by chr22 and ancient-Z.

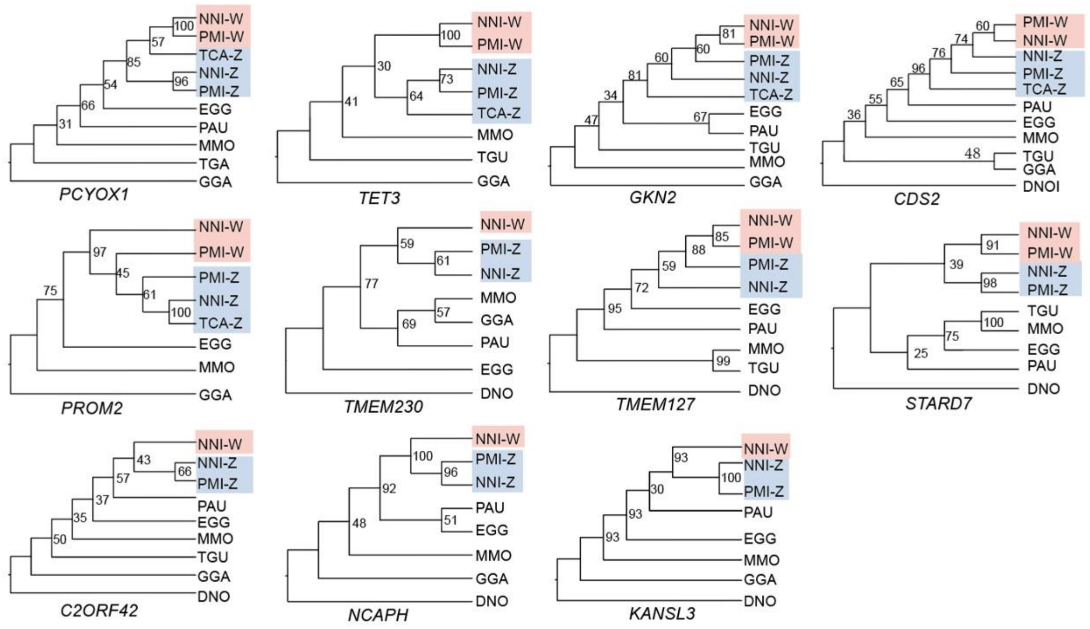

Supplementary Fig. 11 Gene trees for the neo- Z and W linked gametologs of S4. Gene name is shown under each tree. neo-Z and neo-W linked gametologs of crested ibis are highlighted in blue and red, respectively. EGG (egret, *egretta garzetta*, belongs to Ardeidae family). Threskiornithidae genes are grouped by sex chromosome rather than species.

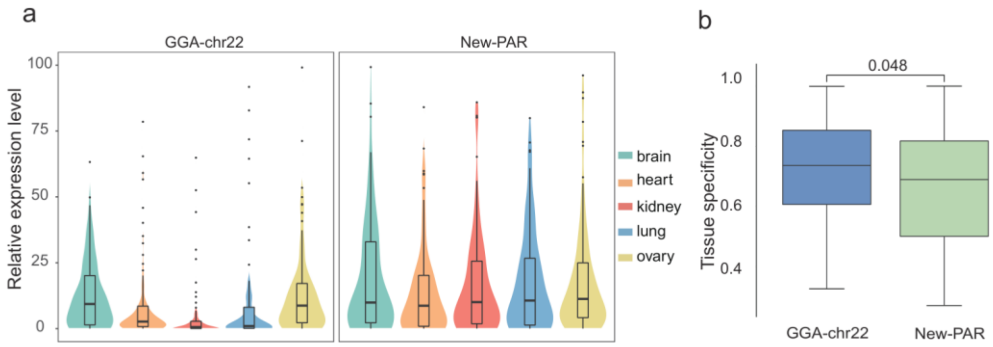

Supplementary Fig. 12 Shifted gene expressional patterns of ASCRs. a The expression for New-PAR genes and their orthologs of chr22 of chicken in different tissues. **b** The expression tissue specificity (tested by Tau value) for New-PAR genes and their orthologs of chr22 of chicken. Two-sided T-test. Number on the horizontal line represents p value. Source data are provided as a Source Data file.

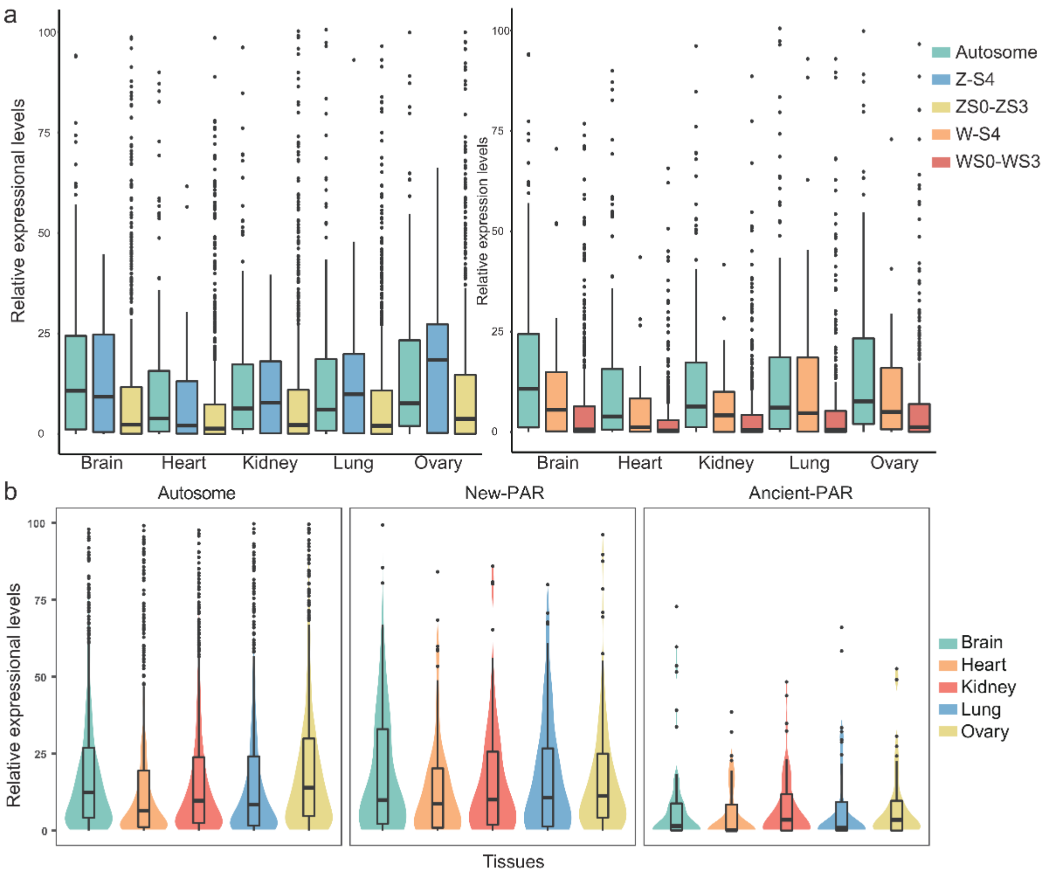

Supplementary Fig. 13 Comparisons of expression levels between ancient and new region on sex chromosomes in five tissues. a Comparison between autosome, neo-Z-S4, neo-ZS0-S3, neo-W-S4 and neo-WS0-S3. n=894, 28, 943, 28 and 447 genes of autosomes (chr3 was taken as an example), Z-S4, ZS0-ZS3, W-S4 and WS0-WS3, respectively. **b** Comparison between autosome, new-PAR and ancient-PAR. n=894, 94 and 46 genes of autosome (chr3), new-PAR and ancient-PAR, respectively. Source data are provided as a Source Data file.

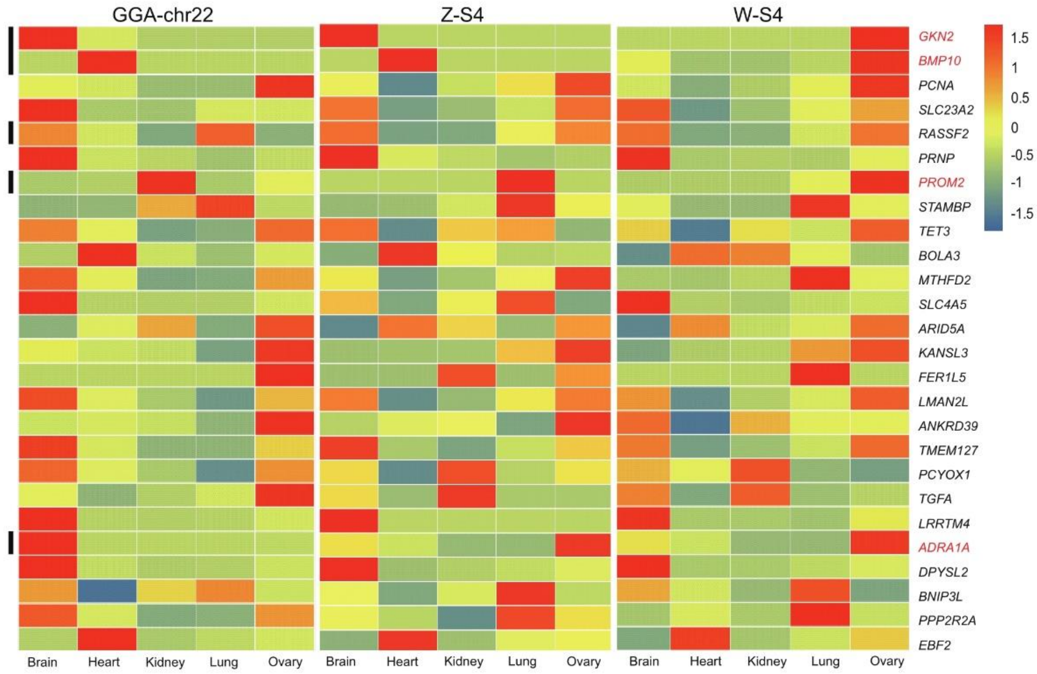

Supplementary Fig. 14 A heatmap showing the expression (measured by $\log_2$FPKM) of S4 genes on neo-Z/W and their orthologs of chr22 of chicken (GGA) in different tissues. The left panel shows the expressions of the orthologs in chr22 of chicken in five tissues. The medium and right panel show the expressions of S4 genes locate on neo- Z and W of crested ibis in five tissues respectively. Each row represents one ortholog. The expression level is shown by log-transformed FPKM values as color-coded heatmap. The gene that marked in red indicates an ovarian biased shift in expression from chr22 in chicken to sex chromosomes.

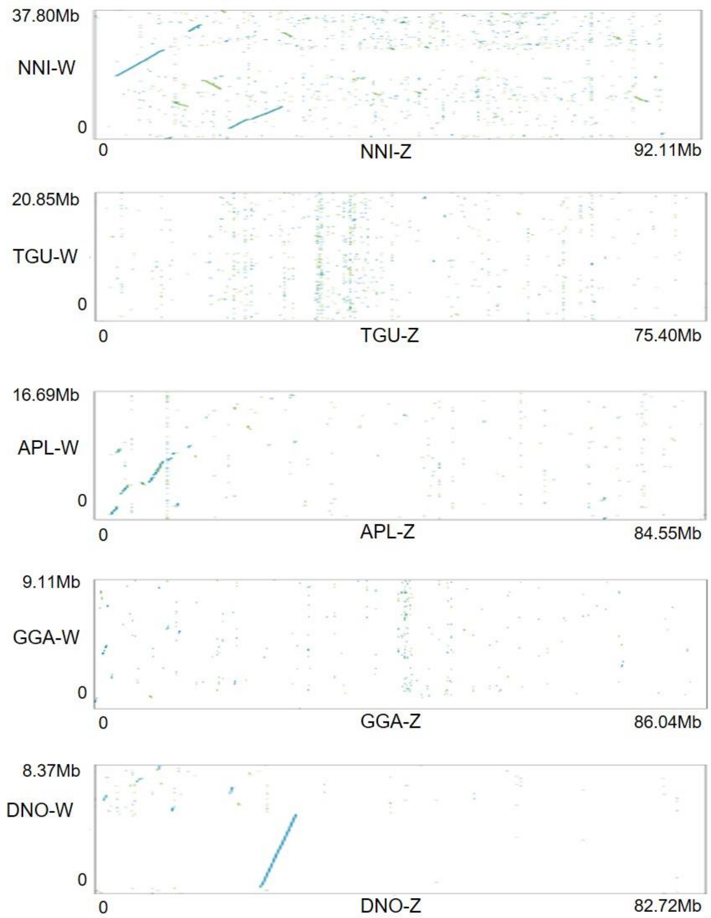

Supplementary Fig. 15 Comparison of ZW synteny between different species. NNI showed highly conserved synteny blocks, almost no synteny block of Z and W exhibited in GGA (*Gallus gallus*) and TGU (*Taeniopygia guttata*).

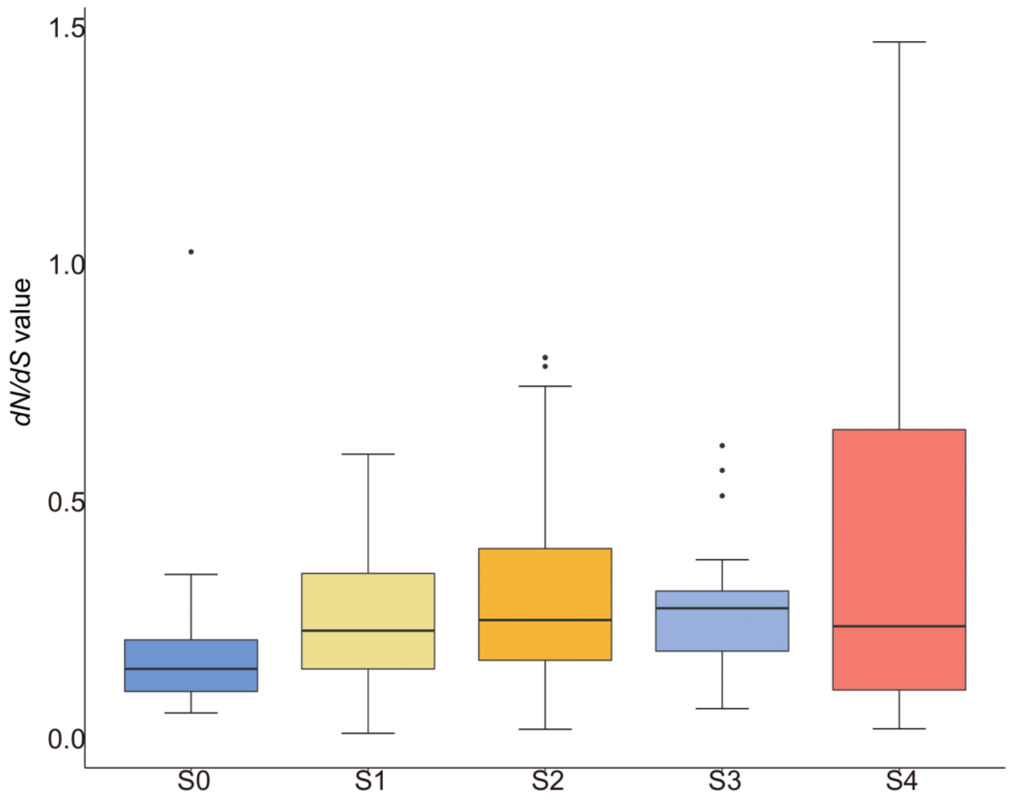

Supplementary Fig. 16 Comparing of *dN/dS* value of genes in different strata. There is no significant difference between the *dN/dS* value of S4 and the ancient stratum (S0-S3). n=13, 29, 79, 21, 32 of S0, S1, S2, S3 and S4, respectively. Source data are provided as a Source Data file.

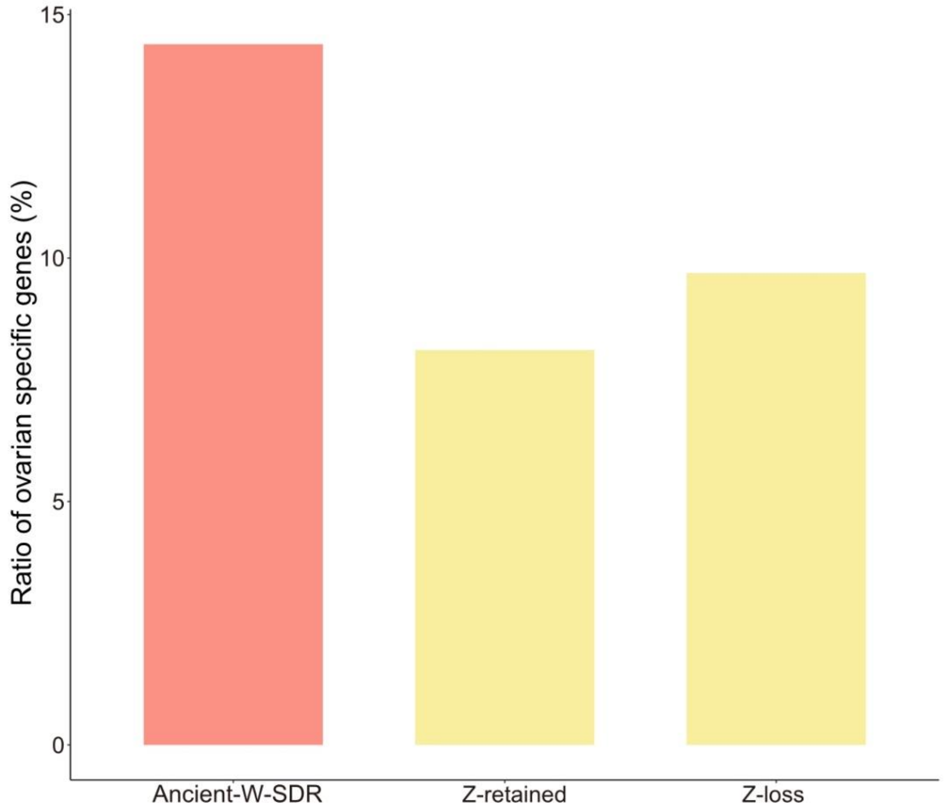

Supplementary Fig. 17 Comparison of ratio of ovarian specific genes in sex chromosomes. The red and yellow columns represent SDRs on ancient- W and Z chromosomes respectively. Z-retained: the ancient-Z-linked genes with W-linked homologues retained**.** Z-loss: the ancient-Z-linked genes whose W-linked homologues have lost.

Supplementary Tables

Supplementary Table 1 Information of the sequencing data of crested ibis generated by this research.

Read type	Gender	Tissue	Sample ID	Total Data (Gb)	Depth (x)	Mapping rate (%)
Pacbio-HiFi	female	Muscle	580	48	37	99.95
Hi-C	female	Muscle	580	239	184	99.91
Resequencing	female	Blood	096	24	19	97.43
Resequencing	female	Blood	099	27	21	98.13
Resequencing	female	Blood	710	27	21	97.36
Resequencing	male	Blood	094	26	20	97.21
Resequencing	male	Blood	100	28	22	98.09
Resequencing	male	Blood	668	27	21	98.12
RNA-seq	female	Heart	580	8	7	97.34
RNA-seq	female	Brain	580	8	7	96.14
RNA-seq	female	Lung	580	8	7	95.68
RNA-seq	female	Kidney	580	8	7	96.81
RNA-seq	female	Ovary	580	8	7	95.15

Supplementary Table 2 The assembly evaluation of genomes of crested ibis and six published birds

	Crested ibis (this study)	Emu	Chicken	Mallard	Golden eagle	Zebra finch	Monk parakeet
Chromosome level genome size (Gb)	1.31	1.26	1.05	1.19	1.23	1.06	1.17
N50 contig (Mb)	16.39	13.3	18.8	5.7	21.9	9	24.5
N50 scaffold (Mb)	102.8	82.7	90.9	76.3	46.9	81	75.7
BUSCO complete (%)	97.7	97.0	96.6	96.7	97.4	96.5	96.7

Supplementary Table 3 The statistics and characteristics of each chromosome in the crested ibis genome

Chromosome	Chromosome Size(bp)	Anchored contig number	Anchored gene number	GC content (%)
Chr1	171562090	24	1738	41
Chr2	132730213	18	1465	40.8
Chr3	130242979	10	1571	41.4
Chr4	128447226	17	1926	41.6
Chr5	102769032	4	1788	42.5
Chr6	85029433	3	1045	40.7
Chr7	72926767	11	1219	42
Chr8	56183815	4	1062	43
Chr9	50865095	3	983	43.5
Chr10	25313139	2	529	44.1
Chr11	22656060	2	501	45.2
Chr12	17152688	4	464	46.2
Chr13	16606332	4	476	46.5
Chr14	14594511	3	429	47.3
Chr15	13544887	1	425	47.3
Chr16	13144920	1	407	48.2
Chr17	9847061	4	327	47.6
Chr18	8651685	2	338	50.8
Chr19	8469463	1	242	49.9
Chr20	8192294	7	412	52.4
Chr21	7743769	3	363	52
Chr22	6498024	3	378	53.7
Chr23	3737770	2	386	58.4
Chr24	3466903	13	311	59.9
Chr25	1415944	10	151	62.1
Chr26	818494	8	76	62.3
Chr27	618678	5	78	59.8
Neo-W-SDR	37804327	24	414	43.3
Neo-Z	91676785	30	1112	41.3

Supplementary Table 4 Proportions of pseudogenes in different regions of crested ibis genome

Region	Pseudogene-num	Total	Pseudo-ratio
WS4	4	75	5.33%
WS0-3	36	339	10.59%
ZS4	2	53	3.77%
ZS0-3	15	859	1.75%
Neo-PAR	8	154	5.19%
Ancient-PAR	0	46	0.00%
Autosomes	3324	16734	19.86%

Supplementary Table 5 Statistics of characters of genes on ancient-W

	Total	Num of pseudogene
Single-copy	206	15
Multi-copy	133	21
Sum	339	36

Supplementary Table 6 GO enrichment of genes on ancient-SDR of the neo-W chromosome

GO ID	GO-term name	P-Value	Corrected P-Value
GO:0008284	positive regulation of cell population proliferation	1.53E-06	0.001101578
GO:0000902	cell morphogenesis	0.000107	0.036168765
GO:0043524	negative regulation of neuron apoptotic process	0.00021	0.036168765
GO:2000727	positive regulation of cardiac muscle cell differentiation	0.000265	0.036168765
GO:0046549	retinal cone cell development	0.000353	0.036168765
GO:0032790	ribosome disassembly	0.000353	0.036168765
GO:0004896	cytokine receptor activity	0.000434	0.036168765
GO:0030154	cell differentiation	0.00045	0.036168765
GO:0046548	retinal rod cell development	0.000453	0.036168765
GO:0004652	polynucleotide adenylyltransferase activity	0.000565	0.039507344
GO:0003139	secondary heart field specification	0.000688	0.039507344
GO:0060993	kidney morphogenesis	0.000688	0.039507344
GO:0005794	Golgi apparatus	0.000746	0.039507344
GO:0071044	histone mRNA catabolic process	0.000824	0.039507344
GO:0060766	negative regulation of androgen receptor signaling pathway	0.000824	0.039507344

Statistical test used for data analysis was Fisher's exact test with correction method: Benjamini and Hochberg.

Supplementary Table 7 Ovarian bias ratio of two classes of genes on the ancient-SDR of the neo-W chromosome of crested ibis

Gene class	Ovary-biased num	Total num	Ratio of ovarian-biased genes
Birds-shared	6	103	5.82%
NNI-specific-retained	27	237	11.39%
