# Supplementary material for: Evolution and expression patterns of the neo-sex chromosomes of the crested ibis
Source: Nat Commun. 2024 Feb 23;15:1670. doi: 10.1038/s41467-024-46052-x (PMC10891136; doi:10.1038/s41467-024-46052-x)
Supplement: Supplementary file 3 — Reporting Summary [file 41467_2024_46052_MOESM3_ESM.pdf]

nature portfolio

Corresponding author(s): Gang Li, Xiaoping Yu, Luohao Xu

Last updated by author(s): 2024-1-22

Reporting Summary

Nature Portfolio wishes to improve the reproducibility of the work that we publish. This form provides structure for consistency and transparency in reporting. For further information on Nature Portfolio policies, see our Editorial Policies and the Editorial Policy Checklist.

Statistics

For all statistical analyses, confirm that the following items are present in the figure legend, table legend, main text, or Methods section.

n/a	Confirmed	
☐	☒	The exact sample size (n) for each experimental group/condition, given as a discrete number and unit of measurement
☐	☒	A statement on whether measurements were taken from distinct samples or whether the same sample was measured repeatedly
☐	☒	The statistical test(s) used AND whether they are one- or two-sided<br>*Only common tests should be described solely by name; describe more complex techniques in the Methods section.*
☐	☒	A description of all covariates tested
☐	☒	A description of any assumptions or corrections, such as tests of normality and adjustment for multiple comparisons
☐	☒	A full description of the statistical parameters including central tendency (e.g. means) or other basic estimates (e.g. regression coefficient) AND variation (e.g. standard deviation) or associated estimates of uncertainty (e.g. confidence intervals)
☐	☒	For null hypothesis testing, the test statistic (e.g. F, t, r) with confidence intervals, effect sizes, degrees of freedom and P value noted<br>*Give P values as exact values whenever suitable.*
☒	☐	For Bayesian analysis, information on the choice of priors and Markov chain Monte Carlo settings
☐	☒	For hierarchical and complex designs, identification of the appropriate level for tests and full reporting of outcomes
☐	☒	Estimates of effect sizes (e.g. Cohen's d, Pearson's r), indicating how they were calculated

Our web collection on statistics for biologists contains articles on many of the points above.

Software and code

Policy information about availability of computer code

Data collection	No softwares were used for Data collection.
Data analysis	Hifiasm (v0.13), Juicer (v1.6), 3D-DNA (v201008), Juicebox(1.11.08), SOAPdenovo version2.04, Tandem Repeats finder (4.09), RepeatProteinMasK version 1.23(RM-BLASTX), RepeatModeler2(2.0), RepeatMasker (open-4.0.7), parseRM pipeline (https:/ github.com/4ureliek/Parsing-RepeatMasker-Outputs),Augustusl version 2.5.5, blast+(2.10.1), Genewise v2.4.1, BLAT version 34, PASA version 2.1.0, EvidenceModeler v1.1.1, MUMmer (4.0.0.beta2), MCscan JCVI utility pipeline v1.3.9, BWA (version 0.7.17-r1188), Samtools-1.9, Picard Tools (Version 1.56), GATK(version 3.8), minimap2-2.17, IGV(2.11.3), LASTZ (1.04), MEGA-CC 11, Orthofinder (version 2.5.4), MAFFT (v7.475), PRANK (v.170427), PAML package (v.4.9e), RAxML (V. 7.0.4), HISAT2 (2.2.0), RSEM pipeline (v.1.3.0), STAR (v.2.5.30). Scripts and pipelines used for sex chromosomes analysis were downloaded from https:/github.com/lurebgi/BOPsexChr.

For manuscripts utilizing custom algorithms or software that are central to the research but not yet described in published literature, software must be made available to editors and reviewers. We strongly encourage code deposition in a community repository (e.g. GitHub). See the Nature Portfolio guidelines for submitting code & software for further information.

Data

Policy information about availability of data

All manuscripts must include a data availability statement. This statement should provide the following information, where applicable:

- Accession codes, unique identifiers, or web links for publicly available datasets
- A description of any restrictions on data availability
- For clinical datasets or third party data, please ensure that the statement adheres to our policy

The assembled chromosome-leveled reference genome of the crested ibis has been deposited in the GenBank with the assembly accession ID of GCA_035839065.1 [https://www.ncbi.nlm.nih.gov/assembly/GCA_035839065.1/?shouldredirect=false]. The project accession number of this work that can be accessed in NCBI database is PRJNA974878 [https://www.ncbi.nlm.nih.gov/sra/?term=PRJNA974878], and all raw sequences data generated by the research have been deposited in NCBI database with the SRA accession numbers from SRX20466107 to SRX20466118, and SRX22371138. KEGG database [https://www.genome.jp/kegg/], Gene Ontology database [https://www.geneontology.org/] and Repbase database (Release 16.10) [https://www.girinst.org/repbase/index.html] were used in this work. A full list of download data accession IDs is available in the Supplementary Data 9. Source data are provided as a Source Data file.

Research involving human participants, their data, or biological material

Policy information about studies with human participants or human data. See also policy information about sex, gender (identity/presentation), and sexual orientation and race, ethnicity and racism.

Reporting on sex and gender	No human participants or human data were involved in this study.
Reporting on race, ethnicity, or other socially relevant groupings	No human participants or human data were involved in this study.
Population characteristics	No human participants or human data were involved in this study.
Recruitment	No human participants or human data were involved in this study.
Ethics oversight	No human participants or human data were involved in this study.

Note that full information on the approval of the study protocol must also be provided in the manuscript.

Field-specific reporting

Please select the one below that is the best fit for your research. If you are not sure, read the appropriate sections before making your selection.

☐ Life sciences ☐ Behavioural & social sciences ☒ Ecological, evolutionary & environmental sciences

For a reference copy of the document with all sections, see nature.com/documents/nr-reporting-summary-flat.pdf

Ecological, evolutionary & environmental sciences study design

All studies must disclose on these points even when the disclosure is negative.

Study description	We used HiFi and Hi-C sequencing technology to assemble a chromosomal level reference genome of a female crested ibis (Nipponia nippon), we analysis the karyotype changes and evolutionary history of sex chromosomes of crested ibis, in addition, we collected RNA-seq data from somatic and gonadal tissues to conduct a comprehensive analysis of global gene expression on the sex chromosomes.
Research sample	To obtain the sequence data of the crested ibis' sex chromosomes, including both Z and W chromosomes, we assembled a high-quality reference genome of a female crested ibis. Tissue samples preserved in liquid nitrogen were applied from Shaanxi Rare Wildlife Rescue Base. These tissues were collected from an adult female crested ibis that had a failed rescue. Blood samples were collected from three adult male and three adult female crested ibis individuals for whole genome resequencing to confirm the assembly of the sex chromosomes. These blood samples were obtained from the Shaanxi Rare Wildlife Rescue Base. As well, to compare the sex chromosomes of the crested ibis with other species of Threskiornithidae, we collected the blood samples of an adult female individual of the black-faced spoonbill from Guangzhou Wildlife Research Center, which was used for whole genome sequencing. To fulfil the genomic comparison analysis, we download the published genome assembly data of emu, chicken, mallard, zebra finch, monk parakeet, golden eagle and double-crested cormorant respectively from NCBI database.
Sampling strategy	Sampling procdure: The blood samples were obtained using the method of brachial wing vein blood collection. The tissues used for RNA-seq were collected from an adult female crested ibis individual that was failed to be rescued. Organ tissue were collected and froze in liquid nitrogen immediately and then transferred to -80 ° C freezer for long-term preservation. Skin sample was taken from a male adult crested ibis individual. Immediately after disinfection, a 0.3 × 0.6 cm2 area of skin tissue was placed into culture medium. The animal was placed back into the feeding net after bandaging. One individual is sufficient to perform reference genome assembly. The re-sequencing data was only used to calcuate the sequencing depth, three individuals for each sex were sufficient.

Data collection	A 15 Kb DNA SMRTbell library was constructed using a standard protocol for sequencing on the PacBio Sequel II platform with HiFi sequencing to obtain long and accurate raw reads data. The Hi-C library of the crested ibis' genome was constructed by digesting cross-linked chromatin with the restriction enzyme Dpn Ⅱ and sequenced on the Illumina NovaSeq 6000 platform. To obtain resequencing data, DNA libraries were generated for each bird individual and sequenced on the Illumina NovaSeq 6000 platform. For RNA-seq, after extracting mRNA from each tissue, fragmentation buffer was added to randomly break the mRNA. Subsequently, reverse transcription and PCR were executed to construct the library, which was then sequenced on the Illumina NovaSeq 6000 platform.
Timing and spatial scale	These crested ibis samples for sequencing (HiFi, Hi-C, Illumina, RNA-seq) were collected in April 6th 2021 in Xi'an , China. The skin sample was collected in August 20th 2023 in Xi' an, China. The blood sample of black-faced spoonbill was collected in Guangzhou Zoo, Xi'an, China.
Data exclusions	No data were excluded.
Reproducibility	We collected the blood samples from three male and three female individuals. All attempts to repeat the experiments were successful.
Randomization	The individuals involved in this study were randomly selected.
Blinding	All sampled animals were raised in similar conditions in the same animal facility, so no blinding was needed

Did the study involve field work? ☐ Yes ☒ No

Reporting for specific materials, systems and methods

We require information from authors about some types of materials, experimental systems and methods used in many studies. Here, indicate whether each material, system or method listed is relevant to your study. If you are not sure if a list item applies to your research, read the appropriate section before selecting a response.

Materials & experimental systems

n/a	Involved in the study	
☒	☐	Antibodies
☐	☒	Eukaryotic cell lines
☒	☐	Palaeontology and archaeology
☐	☒	Animals and other organisms
☒	☐	Clinical data
☒	☐	Dual use research of concern
☒	☐	Plants

Methods

n/a	Involved in the study	
☒	☐	ChIP-seq
☒	☐	Flow cytometry
☒	☐	MRI-based neuroimaging

Eukaryotic cell lines

Policy information about cell lines and Sex and Gender in Research

Cell line source(s)	A skin sample was taken from a male adult crested ibis individual for cell line culture.
Authentication	HE staining showed that the cells were spindle or polygonal in shape, with a red cytoplasm and a blue nucleus, which are consistent with the morphological characteristics of fibroblasts. The identification of vimentin showed that the cytoplasm was stained brown, which is a specific staining of vimentin, indicating no epithelial cells contamination.
Mycoplasma contamination	Cell line tested negative for mycoplasma contamination.
Commonly misidentified lines (See ICLAC register)	No commonly misidentified cell lines were used in the study.

Animals and other research organisms

Policy information about studies involving animals; ARRIVE guidelines recommended for reporting animal research, and Sex and Gender in Research

Laboratory animals	The study did not involve laboratory animals.
Wild animals	For crested ibis, tissue samples used in this research for the HiFi, Hi-C and RNA-seq were collected from an adult female individual that had a failed rescue. Blood samples used for resequencing were collected from six captive individuals. Skin samples used for cell lines culturing were collected from a female captive individual. All crested ibis samples were collected from captive individuals bred in Shaanxi Rare Wildlife Rescue Base. The black-faced spoonbill blood sample was collected from an adult captive individual bred in Guangzhou Wildlife Research Center. The animals were placed back into the feeding net after sampling. No animals perished in the

	course of this study (except for a rescue failure individual who died before sampling).
Reporting on sex	For assembling both Z and W sex chromosomes of crested ibis, we collected the tissues samples from a female crested ibis for wholegenome sequencing and assembly. To evaluate the accuracy of the sex chromosome assembly, we collected the blood samples fromextra 3 male and 3 female crested ibis for resequencing works, and the information of sex and sequence data are listed in the Supplementary Table1.
Field-collected samples	This study did not involve samples collected from the field.
Ethics oversight	The study protocols received ethical approval from the Ethics Committee of the Guangzhou Wildlife Research Center (permit number: GZZOO2021C02) and Shaanxi Rare Wildlife Rescue Base (permit number: SRWRB202102). All experimental procedures were approved by the Animal Care and Use Committee of Shaanxi Normal University following the guidelines outlined in the Guide for the Care and Use of Laboratory Animals in China.

Note that full information on the approval of the study protocol must also be provided in the manuscript.

Plants

Seed stocks	No plants sample or data were involved in this study.
Novel plant genotypes	No plants sample or data were involved in this study.
Authentication	No plants sample or data were involved in this study.
