# Supplementary material for: Evolution and expression patterns of the neo-sex chromosomes of the crested ibis
Source: Nat Commun. 2024 Feb 23;15:1670. doi: 10.1038/s41467-024-46052-x (PMC10891136; doi:10.1038/s41467-024-46052-x)
Supplement: Supplementary file 4 — Description of Additional Supplementary Files [file 41467_2024_46052_MOESM4_ESM.pdf]

Description of Additional Supplementary Files

Supplementary Data 1
Description: Statistics of assemblies of W chromosomes in birds uploaded to public database.

Supplementary Data 2
Description: The results of alignments between the crested ibis' neo-Z chromosome and chicken's genome.

Supplementary Data 3
Description: The results of alignments between the crested ibis’ neo-W chromosome and chicken's genome.

Supplementary Data 4
Description: List of identified one-to-one gametologs between the neo-Z and W chromosomes of crested ibis.

Supplementary Data 5
Description: Identified one to one orthologs among chromosome 22 of chicken, neo-W and neo-Z chromosomes of crested ibis.

Supplementary Data 6
Description: Genes retained in the ancient-SDR of the neo-W chromosome of crested ibis.

Supplementary Data 7
Description: The expression profiles of ovary specific genes on ancient-SDR of the neo-W chromosome of crested ibis.

Supplementary Data 8
Description: Chicken chr22 Sequences and primers for specific probes synthesis.

Supplementary Data 9
Description: Accessions of Genome and RNA data of other species.by a two-serial MEG bank.
